# Real-Life Data of 2-Year Lumasiran Use in the DAILY-LUMA Cohort

**DOI:** 10.1016/j.ekir.2024.12.033

**Published:** 2024-12-30

**Authors:** Anne-Laure Sellier-Leclerc, Melissa Cloarec, Bertrand Knebelmann, Lise Allard, Olivia Boyer, Sylvie Cloarec, Claire Dossier, Moglie Le Quintrec, François Nobili, Thomas Stehlé, Isabelle Vrillon, Stéphane Burtey, Emilie Cornec-Le Gall, Marie Courbebaisse, Thierry Frouget, Arnaud Garnier, Thierry Krummel, Sandrine Lemoine, Catherine Monet-Didailler, Caroline Rousset-Rouvière, Amélie Ryckewaert, Adeline Schendel, Sacha Flammier, Cécile Acquaviva-Bourdain, Justine Bacchetta

**Affiliations:** 1 Reference Center for Rare Renal Diseases, ORKID and ERK-Net networks, Lyon University Hospital, Lyon, France; 2 Nephrology and Transplantation Department, Centre de référence MAladies Rénales Héréditaires de l’Enfant et de l’Adulte, Hôpital Necker-Enfants malades, AP-HP, Université de Paris, Paris, France; 3 Pediatric Nephrology Unit, Centre de reference constitutif des maladies rénales rares, CHU de Bordeaux, Bordeaux, France; 4 Pediatric Nephrology Unit, Centre de Référence Maladies Rénales Héréditaires de l'Enfant et de l'Adulte, Imagine Institute, Paris Cité University, Hôpital Necker-Enfants malades, AP-HP, Paris, France; 5 Pediatric Nephrology Unit, CHU Tours, Tours, France; 6 Pediatric Nephrology Department, Hôpital Robert Debré, AP-HP, Paris, France; 7 Department of Nephrology, CHU de Montpellier, Université de Montpellier, Montpellier, France; 8 Pediatric Nephrology Unit, CHU Besançon, Besançon, France; 9 Institut National de la Santé et de la Recherche Médicale U955, Institut Mondor de Recherche Biomédicale, Universite Paris Est Créteil, Créteil, France; 10 Innovative therapy for immune disorders, Fédération Hospitalo-Universitaire, Assistance Publique des Hôpitaux de Paris, Hôpitaux Universitaires Henri Mondor, Service de Néphrologie et Transplantation, Créteil, France; 11 Pediatric Unit, hôpital d’enfants, CHRU de Nancy, France; 12 Centre de néphrologie et transplantation rénale, Hopital de la Conception, AP-HM, France; 13 Aix Marseille Univ, INSERM, INRAE, C2VN, Marseille, France; 14 Génétique, Génomique fonctionnelle et Biotechnologies, Unité Mixte de Recherche 1078, Université de Bretagne Occidentale, Inserm, Brest, France; 15 Service de Néphrologie, Centre de Référence Maladies Rénales Héréditaires de l'Enfant et de l'Adulte, Centre Hospitalier Universitaire Brest, Brest, France; 16 Physiology Department, Hôpital européen Georges-Pompidou, APHP, Paris, France; 17 Nephrology Department, CHU de Pontchaillou, Rennes, France; 18 Pediatric Unit, CH de Raiatea, Raiatea, France; 19 Nephrology Department, CHRU de Strasbourg, Strasbourg, France; 20 Pediatric Unit, Centre Hospitalier de La Côte Basque, Bayonne, France; 21 Assistance Publique des Hôpitaux de Marseille, Pediatric Nephrology Unit, Hôpital La Timone, Marseille, France; 22 Pediatric Nephrology Unit, Hospital of Rennes, Rennes, France; 23 Nephrology Department, CH Simone Veil, Troyes, France; 24 Inborn Errors of Metabolism Unit, Biochemistry and Molecular Biology Department, Lyon University Hospital, Bron, France; 25 Université Paris Cité, Paris, France; 26 INSERM1033 Research Unit, Lyon, France

**Keywords:** dialysis, hyperoxaluria type 1, lumasiran, real-life data, RNA interference, transplantation

## Abstract

**Introduction:**

Lumasiran is a drug used in RNA-interference (RNAi) therapy for primary hyperoxaluria type 1 (PH1). Data on its efficacy and safety mainly come from industry-sponsored trials.

**Methods:**

For postmarketing follow-up, French authorities requested a quasi-exhaustive retrospective and prospective study over 5 years for patients receiving lumasiran, requiring the inclusion of at least 90% of patients, named as the DAILY-LUMA cohort (NCT06225882). Here, we analyzed data from all patients who were not previously included in the industry-sponsored trials and had received lumasiran for at least 2 years.

**Results:**

We included 38 patients, 22 from DAILY-A (i.e., estimated glomerular filtration rate (eGFR) > 45 ml/min per 1.73 m^2^, age ≥ 6 years), 6 from DAILY-B (i.e., eGFR > 45 ml/min per 1.73 m^2^, age < 6 years), and 10 from DAILY-C (i.e., all ages, eGFR < 45 ml/min per 1.73 m^2^, 6 on dialysis). In DAILY-A and DAILY-B, decreased urinary oxalate-to-creatinine (UOx/creat) ratio, stable eGFR, and decrease in both nephrocalcinosis severity and stone numbers were observed, with a progressive tapering of conservative therapies. The decreased proportion of patients with nocturnal hydration and G-tubes overtime likely reflects improved quality of life. With a low number of patients — 2 patients on peritoneal dialysis and 3 patients with infantile oxalosis — the results are less conclusive for DAILY-C; however, in older patients, change in plasma oxalate (POx) levels is similar to previously published data. Tolerance was good with no severe side effects; injection site reactions, abdominal pain, and headaches were the main adverse events.

**Conclusion:**

DAILY-LUMA is the largest cohort of patients receiving lumasiran in real life, confirming its safety and efficacy at 2 years.

## Graphical abstract

**Figure:**
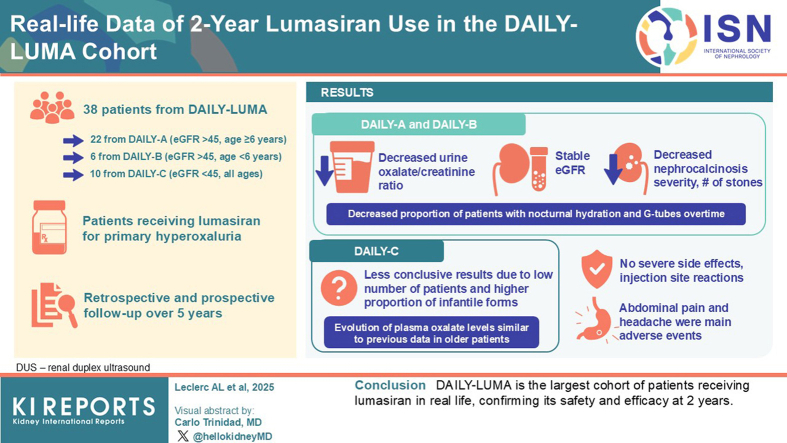

PH1 is due to a mutation in the *AGXT* gene, encoding the hepatic peroxisomal enzyme, alanine-glyoxylate aminotransferase (AGT). Defects in AGT increase the production of oxalate (a product with low solubility), further inducing nephrocalcinosis and kidney stones.^1^^,^^2^ There is a wide spectrum of disease severity, from recurrent bilateral kidney stones with moderate chronic kidney disease (CKD) in adulthood to early kidney failure in the most severe cases of infantile oxalosis.^1^^,^^3^^,^^4^ Until 2020, the treatment of PH1 was nonspecific,^2^ combining urine alkalization, intensive hyperhydration, and conservative CKD management, with the use of pyridoxine in vitamin B6–responsive patients.^5^ However, targeted therapeutic strategies have emerged, using RNAi therapies^6^: lumasiran was approved in the US and in Europe in 2020, whereas nedosiran was approved in the US in 2023. Phase 3 studies showed the efficacy of lumasiran to decrease urinary oxalate (UOx) in patients with moderate or advanced CKD,^7^^,^^8^ and POx levels in advanced CKD and dialysis^9^; middle-term data published at 12 months confirmed the previous results, with decreased stone rates and nephrocalcinosis severity with stable renal function.^10^^,^^11^ In 2023, the OxalEurope consortium and the European Reference Network for Rare Kidney Diseases updated the guidelines for diagnosis and management of PH, notably by delineating more precisely, the use of RNAi in routine treatment.^3^ Recently, novel transplantation strategies combining isolated kidney transplantation with lumasiran have been reported,^12^^,^^13^ with promising results at 2 years.^14^

The question of whether decrease in UOx may slow the change of CKD in PH1 remains open, even though recent data from the OxalEurope registry clearly show an association between UOx and onset of kidney failure in 932 patients with PH1.^15^ Moreover, RNAi therapies are expensive, and their market approval has been mainly based on intermediate endpoints, such as UOx and POx. Lastly, the safety profile of the first approved RNAi therapy (i.e., patisiran in 2018) is reassuring^16^; however, long-term data both in terms of efficacy and safety of lumasiran remain to be evaluated. With all these data in mind, the French Drug Regulatory Agency (also known as Haute Autorité de Santé) has approved the use of lumasiran, provided that the pharmaceutical company is able to provide a 5-year follow-up for at least 90% of patients receiving lumasiran in France. This request was made directly to the company, which in turn requested the Reference Center for Rare Renal Diseases of Lyon to conduct the study with a financial support to perform it, but without any involvement in the design, data analysis and reporting.

The DAILY-LUMA study was therefore designed to provide real-life data on 5-year follow-up of lumasiran use in PH1 in France. Here, our objective is to present data on efficacy and safety assessment at 2 years in a nationwide real-life setting.

## Methods

The DAILY-LUMA study (NCT06225882) was approved by the institutional review board (Comité d’Ethique des Hospices Civils de Lyon, session 22/7/2022, approval number 22-698). The study design is retrospective for the years 2020 to 2023 and prospective for the years 2023 to 2026; the prospective follow-up was harmonized (i.e., with a proposed follow-up) within all centers (but not standardized to respect the “daily-life” setting), but the retrospective part consisted of collecting existing data without standardized management within centers.

As per the authorization by the European Medicine Agency, the protocol for lumasiran administration was the following: patients with weight < 10 kg receive monthly injections (i.e., 6 mg/kg 4 times followed by 3 mg/kg every month). Patients with weight > 10 kg receive quarterly injections after the loading phase (i.e., 6 mg/kg monthly for 4 months, followed by 6 mg/kg every 3 months in patients with weight between 10 and 20 kg; and 3 mg/kg monthly for 4 months, followed by 3 mg/kg every 3 months in patients with weight > 20 kg).

The objective of this study was to provide data with deep granularity. The main data collected included the following: clinical data (age at first symptoms, age at diagnosis, disease activity as defined by lithiasis, nephrocalcinosis, and/or urinary tract infection), management data (conservative therapy of PH1 with compounds and duration, hyperhydration through G-tubes or nasogastric tubes if any, initiation of RNAi therapies, dose of RNAi, safety of RNAi, participation in the patients’ program FREEOSE, dialysis details, transplantation details, and conservative therapy of CKD with a particular focus on the dose of erythropoietin-stimulating agents), genetic data (underlying mutation), pregnancy or lactation (if any), biological data (POx, standard electrolytes, liver function tests, vitamin D and parathyroid hormone levels, blood count, thyroid evaluation, creatine phosphokinase, lactate dehydrogenase, and urinary electrolytes and oxalate on spot and on 24-hour urinary collection if available), and radiological data (renal ultrasounds, bone evaluation through standard X-rays, dual X-ray absorptiometry, or bone computed tomography scan, and cardiac ultrasounds). Data were recorded at baseline, with updates at 1, 2, 3, 6, 9, and 12 months, and every 6 months thereafter for a total duration of 5 years. Data from patients who have been treated under the temporary French authorization for use (since January 2020) and until the implementation of the study were retrospectively collected, and then prospectively followed-up over 5 years; patients newly treated in France between 2020 and 2026 have also been included in DAILY-LUMA. Nocturnal hyperhydration was defined as hyperhydration administered during the night. However, especially in the youngest children, G-Tube may be used not only for hyperhydration but also for medications and feeding. Therefore the p resence of a G-tube does not necessarily mean the presence of nocturnal hyperhydration.

POx and UOx levels were measured using gas chromatography–mass spectrometry method, as previously described.^12^ Reference values adapted to age for UOxwere used notably for the assessment of the UOx/creat ratio; ^3^eGFR was calculated using the 2009 Schwartz equation in children and the 2009 creatinine-based CKD-Epidemiology Collaboration equation in adults.

To ensure an optimal quality of data, we decided to have 1 clinical research associate in charge of data collection for all centers (MCl) based in Lyon and traveling to participating centers. The associate worked closely with an expert physician in PH1 involved in all industry-sponsored trials in PH1 (AL-SL). All data were reviewed by AL-SL.

Here, we focus the analysis on all patients not previously included in industry-sponsored trials that had received lumasiran for at least 2 years. To compare real-life data as much as possible with industry-sponsored trials (namely ILLUMINATE-A, ILLUMINATE-B, and ILLUMINATE-C), and keeping in mind that there was an overlap in eGFR for inclusion criteria between ILLUMINATE-A and ILLUMINATE-C, we decided to divide our present cohort into 3 groups: DAILY-A (i.e., patients aged ≥ 6 years with eGFR > 45 ml/min per 1.73 m^2^; subgroup of pediatric and adult patients), DAILY-B (i.e., patients aged < 6 years, with eGFR > 45 ml/min per 1.73 m^2^), and DAILY-C (i.e., patients of all ages, eGFR < 45 ml/min per 1.73 m^2^, with a subgroup analysis in patients undergoing maintenance dialysis). Of note, only 1 patient included in DAILY-C failed to meet the inclusion criteria in the industry-sponsored trials because of very low UOx levels at screening.

Statistical analyses were performed using the GraphPad Prism software (La Jolla, CA). Descriptive results are presented as median (range). Because of the relative low number of patients in each subgroup, nonparametric Wilcoxon test was performed only between baseline and 2 years. Fisher exact tests was performed to compare proportions.

## Results

### Description of the Cohort

**Figure 1:**
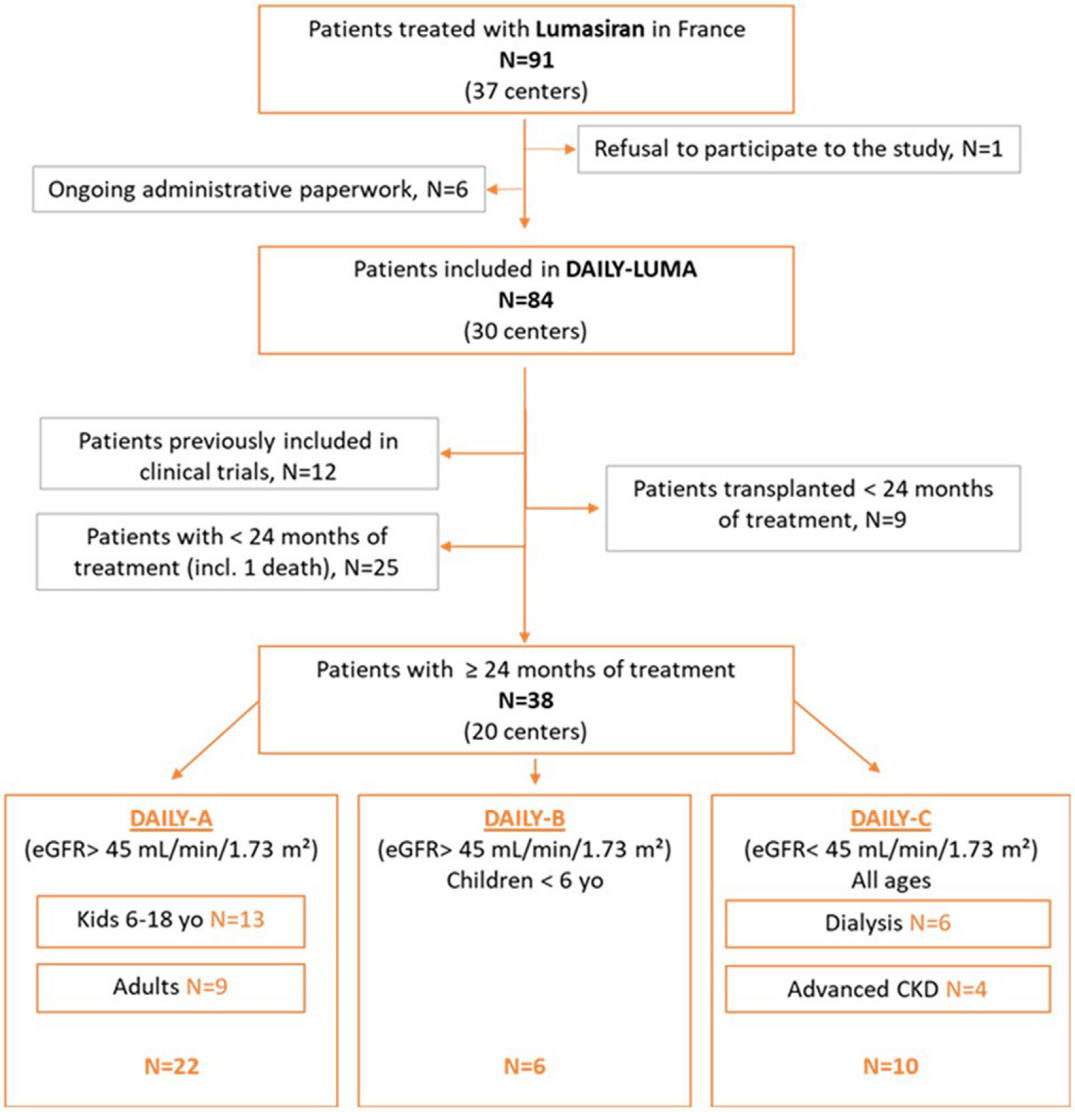
Flow-chart of the study. The numbers were updated on March 31, 2024. CKD, chronic kidney disease; eGFR, estimated glomerular filtration rate.

In total, as of March 31, 2024, 84 patients from 30 different centers were included in the registry. Here, we decided to include all patients having received lumasiran for at least 2 years and who were not previously included in the different industry-sponsored trials, as illustrated in the Flowchart in Figure 1. This corresponds to 38 patients; genotypes are displayed in Supplementary Table S1. Missing visits were reported for 4 patients (1 patient with 2 missing visits, and 3 patients with 1 visit missing). There were no lost-to-follow-up patients; there were 2 deaths in the total cohort (from cancer in both cases) with no impact on the current manuscript (1 patient died before 2 years of follow-up and was not included; the second one died after 2 years of follow-up; the data are available for the current analysis).

### DAILY-A Subcohort (*n* = 22)

**Figure 2:**
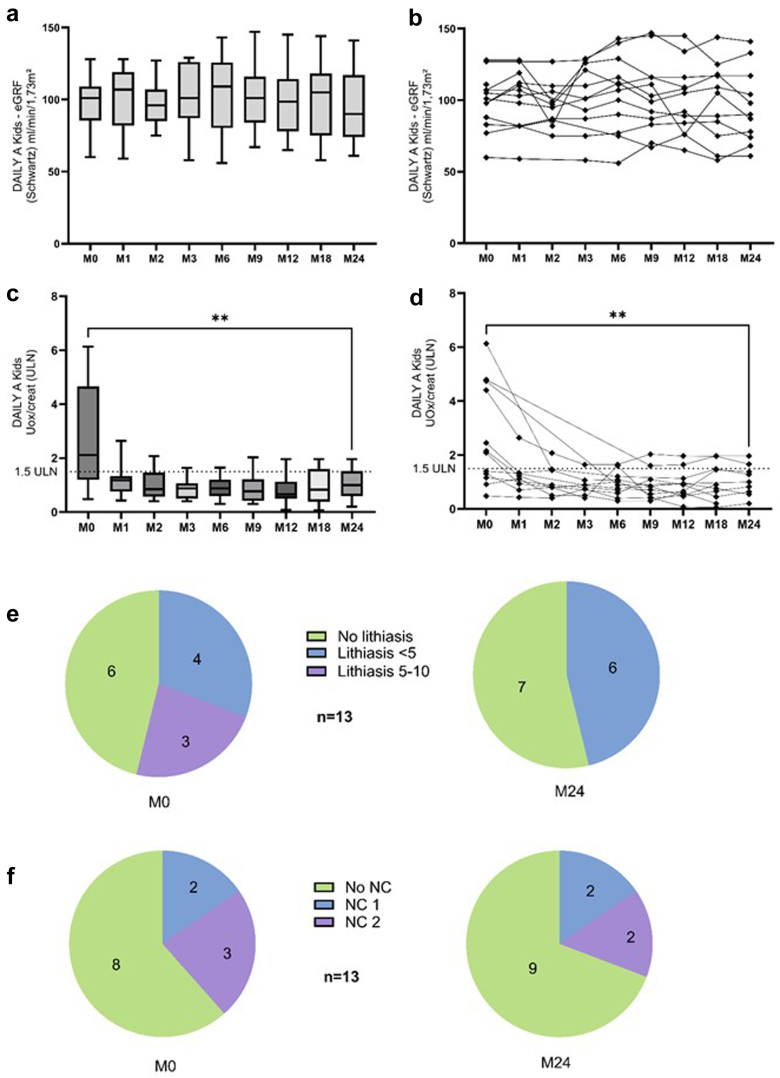
DAILY-A Kids (Children aged 6–18 years, *n* = 13). (a and b) eGFR trends (global view and individual data). (c and d) UOx/creat trends (global view and individual data). (e) Trends in lithiasis numbers on ultrasound imaging. (f) Change in nephrocalcinosis on ultrasound imaging. M0-baseline versus M24 Wilcoxon matched-pairs signed rank test ∗∗*P* < 0.01; UOx/creatinine displayed as xx-times the upper limit for normal (ULN) for age. eGFR, estimated glomerular filtration rate; M, month; NC, nephrocalcinosis; UOx/creat, urinary oxalate-to-creatinine ratio.

The Daily-A subcohort comprised 13 individuals aged between 6 and 18 years at inclusion and 9 aged above 18 years at inclusion. From these patients with conserved kidney function, in Table 1 and Figure 2, we illustrate the main characteristics and trends in the 13 children aged between 6 and 18 years, who started lumasiran at a median age of 13 (6–18) years with a diagnosis at an age of 4.0 (0.1–14.0) years. The proportion of patients receiving nocturnal hydration tended to progressively decrease over time, from 38% at baseline and 8% at 2 years, as well as the proportion of patients with a G-tube (31 to 8%, respectively). Similar trends were observed in terms of the proportion of patients receiving potassium citrate and pyridoxine. Kidney function remained stable over the study period, and individual data of eGFR evaluation even show an improvement of eGFR from 51 to 60 ml/min per 1.73 m^2^ in the patient with the lowest eGFR at lumasiran initiation. UOx/creat ratio significantly decreased between baseline and 2 years; similar to what was observed for eGFR, the 4 patients with the highest UOx/creat ratio at baseline displayed the most important absolute decrease in UOx/creat ratio. Even though results were not significant because of the low number of patients, the severity of nephrocalcinosis decreased, as well as the number of stones on kidney ultrasounds. Three children underwent urological procedures during this 2-year follow-up.

**Table 1:** Results in DAILY-A, pediatric patients

*N* = 13	Parameters	M0 (*n* = 13)	M1 (*n* = 13)	M2 (*n* = 13)	M3 (*n* = 12)	M6 (*n* = 13)	M9 (*n* = 13)	M12 (*n* = 13)	M18 (*n* = 12)	M24 (*n* = 13)	*P*-value (between M0 and M24)
Clinical characteristics at M0	Age (yrs)	13 (6–18)									
	Age at diagnosis (yrs)	4.0 (0.1–14.0)									
	Body weight (kg)	46.8 (17.6–92.7)								55.0 (22.3–91.9)	0.0093
	Body weight (SDS)	0.0 (−1.3 to 3.9)								0.6 (−0.9 to 3.2)	ns
	Height (cm)	151.5 (111.0–174.3)								159.0 (123.0–176.0)	0.0005
	Height (SDS)	0.4 (−1.7 to 1.5)								0.2 (−1.0 to 1.4)	ns
Management	Hydration (l/m^2^/d)	2.0 (1.0–4.0)	2.0 (1.0–4.0)	2.0 (1.0–4.0)	2.0 (1.0–4.0)	2.0 (1.0–4.0)	2.0 (1.0–4.0)	2.0 (1.0–4.0)	3.0 (2.0–6.0)	2.0 (1.0–4.0)	
	Nocturnal hyperhydration (*n*)	5	5	5	5	4	3	2	2	1	ns
	K citrate (*n*)	12	12	12	12	11	11	10	9	9	ns
	K citrate (mg/kg/d)	99 (27–170)	117 (27–176)	113 (27–175)	112 (27–160)	83 (32–208)	83 (32–208)	89 (36–191)	85 (36–180)	83 (32–168)	
	Pyridoxi*n*e (*n*)	10	10	9	8	8	8	7	7	7	ns
	G-tube (*n*)	4	4	4	4	4	4	1	1	1	ns
Biomarkers	eGFR (Schwartz) (ml/min per 1.73 m^2^)	101 (60–128)	107 (59–128)	96 (75–127)	101 (58–129)	109 (56–143)	101 (67–145)	99 (65–114)	105 (58–144)	90 (61–141)	ns
	Bicarbonate (mmol/l)	24.1 (18.0–28.5)	24.6 (20.2–27.1)	24.7 (20.0–28.5)	23.8 (21.6–28.5)	25.0 (22.1–28.7)	23.4 (21.0–26.5)	23.0 (20.0–28.0)	24.0 (21.0–28.0)	25.2 (20.3–27.0)	ns
Urinary biomarkers	Oxalate/creatinine (μmol/mmol)	184 (48–613)	102 (43 –264)	84 (40–207)	74 (40–164)	77 (30–165)	70 (30–203)	60 (8–196)	64 (6–196)	80 (20–196)	0.0039
	Oxalate/creatinine (x-ULN)	2.12 (0.48–6.13)	1.18 (0.43–2.64)	0.85 (0.40–2.07)	0.88 (0.40–1.64)	0.88 (0.30–1.65)	0.77 (0.30–2.03)	0.65 (0.08–1.96)	0.86 (0.06–1.96)	1.00 (0.20–1.96)	0.0039
Renal ultrasounds	Lithiasis (*n*)	7	7	7	7	7	7	7	7	6	ns
	Nephrocalcinosis (*n*)	5	5	5	5	5	5	5	5	4	ns

eGFR, estimated glomerular filtration rate; M, month; NS, nonsignificant *P*; SDS, SD score; x-ULN, x-time the upper limit of normal for age.

Wilcoxon nonparametric test for quantitative variables or Fisher exact test for calculating the proportion of patients receiving hyperhydration/citrate/pyridoxine, etc.

**Figure 3:**
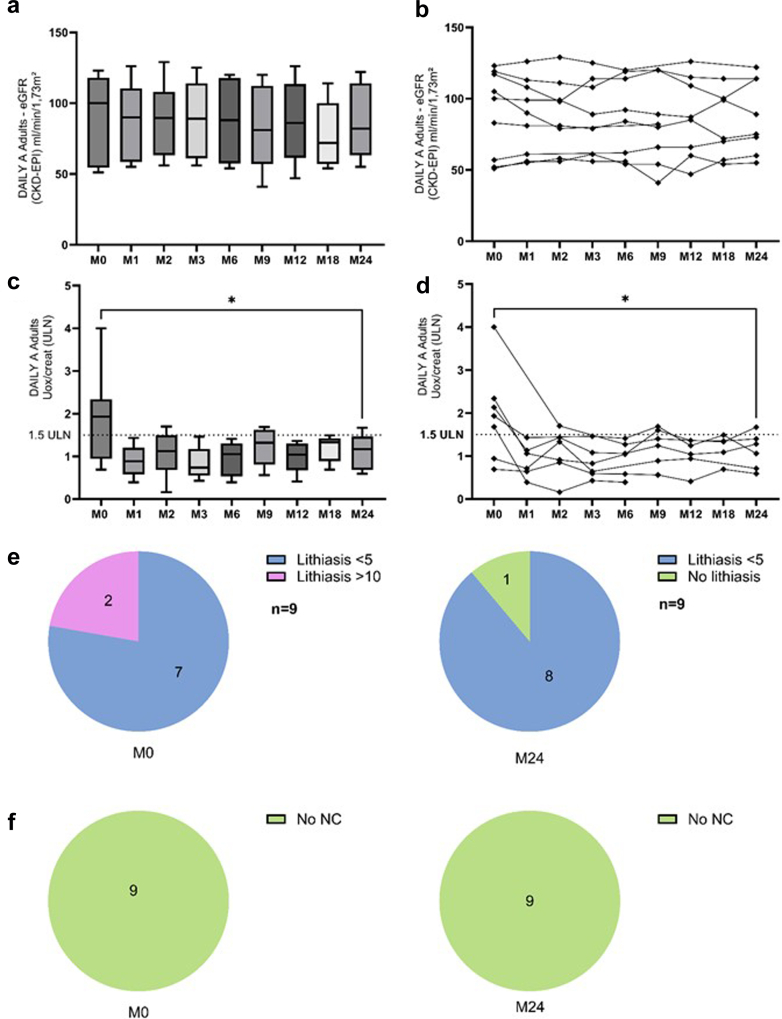
DAILY-A adult patients (Adults aged > 18 years, *n* = 9). (a and b) eGFR trends (global view and individual data). (c and d) UOx/creat trends (global view and individual data). (e) Evolution of lithiasis numbers on ultrasound imaging. (f) Change in nephrocalcinosis on ultrasound imaging. M0-baseline vs. M24 Wilcoxon matched-pairs signed rank test ∗∗*P* < 0.01; UOx/creatinine displayed as xx-times the ULN for age. CKD-EPI, Chronic Kidney Disease Epidemiology Collaboration equation; eGFR, estimated glomerular filtration rate; M, month; NC, nephrocalcinosis; ULN, upper limit for normal; UOx/creat, urinary oxalate-to-creatinine ratio.

The results from the 9 adults from this subgroup are displayed in Table 2 and Figure 3, starting lumasiran at a median age of 31 (19–55) years with a diagnosis at an age of 23 (5–51) years. In contrast to children, there were no obvious changes in the proportion of patients receiving nocturnal hydration, potassium citrate, and pyridoxine. Kidney function remained stable over the study period, and individual data of eGFR evaluation show at least a stabilization of eGFR in the 3 patients with CKD stage 3 at lumasiran initiation. UOx/creat ratio significantly decreased between baseline and 2 years; similar to what was observed in children, the patient with the highest UOx/creat ratio at baseline displayed the most important decrease in UOx/creat ratio. Of note, even though the recommended follow-up in such patients is based on 24-hour UOxe xcretion, this data was not available for analysis because it was rarely performed in real life. Even though results were not significant because of the low number of patients, the number of stones decreased on kidney ultrasounds during the follow-up; there was no nephrocalcinosis. Two adults underwent urological procedures during this 2-year follow-up.

**Table 2:** Results in DAILY-A, adult patients

*N* = 9	Parameters	M0 (*n* = 9)	M1 (*n* = 9)	M2 (*n* = 9)	M3 (*n* = 8)	M6 (*n* = 9)	M9 (*n* = 8)	M12 (*n* = 9)	M18 (*n* = 8)	M24 (*n* = 9)	*P*-value (between M0 and M24)
Clinical characteristics at M0	Age (yrs)	31 (19–55)									
	Age at diagnosis (yrs)	23 (5–51)									
	Body weight (kg)	67.9 (51.0–94.0)								67.1 (51.0–91.0)	ns
	Height (cm)	173 (153–196)								173 (153–196)	ns
	BMI (kg/m^2^)	21 (17–40)									
Management	Hydration (l/m^2^/d)	3.0 (1.5–5.0)	3.0 (1.5–5.0)	3.0 (1.5–5.0)	3.0 (1.5–5.0)	3.0 (1.5–5.0)	3.0 (1.5–5.0)	3.0 (1.5–5.0)	3.0 (1.5–5.0)	3.0 (1.5–5.0)	
	Nocturnal hyperhydration (*n*)	3	2	2	2	2	2	2	2	2	ns
	K citrate (*n*)	7	7	8	8	8	9	9	9	9	ns
	K citrate (mg/kg/d)	33 (21–118)	34 (21–118)	29 (21–118)	29 (21–118)	29 (16–118)	30 (14–118)	31 (14–115)	31 (14–120)	30 (14–118)	
	Pyridoxine (*n*)	4	4	4	4	4	4	4	4	4	ns
	G-tube (*n*)	3	1	1	1	1	1	1	1	1	ns
Biomarkers	eGFR (CKD-EPI) (ml/min per 1.73 m^2^)	100 (51–123)	90 (55–126)	90 (56–129)	89 (56–125)	88 (54–120)	81 (41–120)	86 (47–126)	72 (54–114)	82 (55–122)	ns
	Bicarbonate (mmol/l)	25.1 (23.6–28.4)	27.5 (22.6–28.2)	27.1 (24.7–29.0)	26.0 (21.2–28.4)	26.4 (25.2–30.8)	26.4 (24.1–29.1)	28.3 (21.7–30.0)	27.2 (23.7–29.8)	27.0 (25.7–31.2)	ns
Urinary biomarkers	Oxalate/creatinine (μmol/mmol)	134 (55–187)	71 (31–114)	71 (13–114)	58 (34–117)	64 (31–113)	113 (45–500)	83 (33–109)	87 (40–119)	71 (47–112)	0.0312
	Oxalate/creatinine (x-ULN)	1,54 (0,94–2,34)	1,10 (0,39–1,43)	1,12 (0,16–1,70)	0,95 (0,43–1,46)	1,05 (0,39–1,41)	1,42 (0,89–6,25)	1,04 (0,89–6,25)	1,35 (0,50–1,49)	1,12 (0,63–1,40)	0.0312
Renal ultrasounds	Lithiasis (*n*)	9	9	9	9	8	8	8	8	8	ns
	Nephrocalcinosis (*n*)	0	0	0	0	0	0	0	0	0	

BMI, body mass index; NS, nonsignificant *P*; eGFR: estimated glomerular filtration rate; M, month; NS, nonsignificant *P*; SDS, SD score; x-ULN, x-time the upper limit of normal for age.

Wilcoxon non-parametric test for quantitative variables or Fisher exact test for calculating the proportion of patients receiving hyperhydration/citrate/pyridoxine, etc.

### DAILY-B Subcohort (*n* = 6)

**Figure 4:**
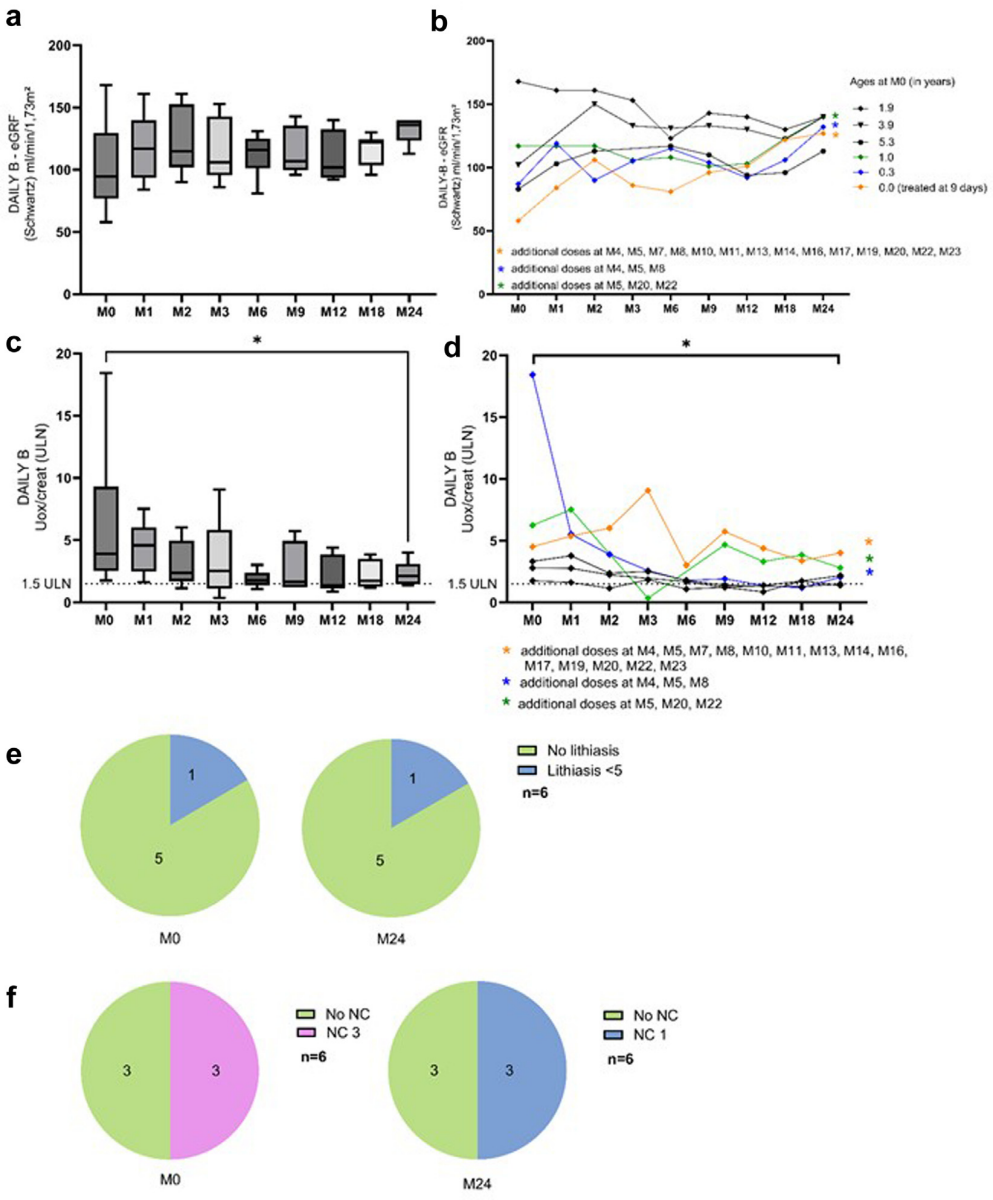
DAILY-B (Children aged < 6 years, *n* = 6). (a and b) eGFR trends (global view and individual data). (c and d) UOx/creat trends (global view and individual data). (e) Change in lithiasis numbers on ultrasound imaging. (f) Change in nephrocalcinosis on ultrasound imaging. Wilcoxon matched-pairs signed rank test M0-baseline vs. M24 ∗*P* < 0.05; UOx/creat displayed as xx-times the ULN for age. eGFR, estimated glomerular filtration rate; M, month; NC, nephrocalcinosis; ULN, upper limit for normal; UOx/creat, urinary oxalate-to-creatinine ratio.

This subgroup consisted of 6 patients aged < 6 years with eGFR > 45 ml/min per 1.73 m^2^, starting lumasiran at a median age of 1.5(0.0–5.0) years with a diagnosis at an age of 0.8 (0.0–2.0) years. In Table 3 and Figure 4, we illustrate the main characteristics and trends. The proportion of patients receiving nocturnal hydration seemed to progressively decrease over time, from 83% at baseline to 67% at 2 years, as well as the proportion of patients with a G-tube (50% to 0%, respectively). Kidney function remained stable over the study period, and individual data of eGFR evaluation even show eGFR improvement in the patient with the lowest eGFR at lumasiran initiation, from 58 to 127 ml/min per 1.73 m^2^ at 2 years; he was aged 9 days at the time of lumasiran initiation. UOx/creat ratio significantly decreased between baseline and 2 years; the patient with the highest UOx/creat ratio at baseline displayed the most important decrease in UOx/creat ratio. Even though results were not significant because of the low number of patients, nephrocalcinosis improved (and even disappeared) as well as the number of stones on kidney ultrasounds. One child underwent urological procedures during this 2-year follow-up. Of note, 3 of 6 patients (with body weights at month 24 of treatment of 10.4, 13.8, and 13.1 kg) have received additional doses of lumasiran, based on physicians’ decision.

**Table 3:** Results in DAILY-B

*N* = 6	Paramteres	M0 (*n* = 6)	M1 (*n* = 6)	M2 (*n* = 6)	M3 (*n* = 6)	M6 (*n* = 6)	M9 (*n* = 6)	M12 (*n* = 6)	M18 (*n* = 6)	M24 (*n* = 6)	*P*-value (between M0 and M24)
Clinical characteristics at M0	Age (yrs)	1.5 (0.0–5.0)									
	Age at diagnosis (yrs)	0.8 (0.3–2.0)									
	Body weight (kg)	9.6 (3.2–24.0)								15.4 (10.4–26.2)	0.0312
	Body weight (SDS)	−0.4 (−1.5 to 1.5)								1.1 (−0.1 to 2.3)	0.0312
	Height (cm)	78.5 (52.0–109.8)								97.8 (83.7–123.0)	0.0312
	Height (SDS)	0.1 (−0.6 to 1.7)								1.9 (−0.2 to 3.0)	ns
Management	Hydration (l/m^2^/d)	1.5 (0.6–2.5)	1.5 (0.7–2.5)	1.5 (1.2–2.5)	1.5 (1.0–1.5)	1.6 (1.2–2.5)	1.5 (0.8–2.5)	1.5 (0.6–2.0)	3.0 (1.5–6.0)	1.6 (1.2–2.0)	
	Nocturnal hyperhydration (*n*)	5	5	5	5	5	5	5	5	4	ns
	K citrate (*n*)	5	5	5	6	6	6	6	6	6	ns
	K citrate (mg/kg/d)	125 (101–241)	123 (101–236)	146 (101–237)	130 (95–237)	133 (113–230)	151 (103–227)	142 (122–207)	133 (72–196)	116 (68–154)	
	Pyridoxine,*n*	5	4	4	4	4	4	4	3	3	ns
	G-tube,*n*	3	3	3	3	2	1	1	1	0	ns
Biomarkers	eGFR (Schwartz) (ml/min per 1.73 m^2^) (> 90)	95 (58–168)	117 (84–161)	115 (90–161)	106 (86–153)	116 (81–131)	107 (96–143)	102 (92–140)	122 (96–130)	136 (113–140)	
	Bicarbonate (mmol/l) (20–28)	21 (18–25)	21 (18–24)	21 (18–24)	21 (19–22)	22 (19–24)	23 (19–24)	23 (19–35)	22 (20–25)	21 (20–23)	
Urinary biomarkers	Oxalate/creatinine (μmol/mmol)	638 (176–2211)	521 (162–1506)	237 (115–1062)	252 (33–1035)	179 (109–302)	184 (123–445)	165 (86–314)	175 (129–366)	177 (105–267)	0.0312
	Oxalate/creatinine (x-ULN)	3.92 (1.76–18.43)	4.59 (1.62–7.52)	2.37 (1.15–4.96)	2.52 (0.35–9.06)	1.78 (1.09–3.02)	1.65 (1.23–5.74)	1.38 (0.86–4.39)	1.75 (1.19–3.85)	2.12 (1.38–4.01)	0.0312
Renal ultrasounds	Lithiasis (*n*)	1	1	1	1	1	2	1	1	1	ns
	Nephrocalcinosis (*n*)	3	3	3	3	3	4	4	4	3	ns

eGFR, estimated glomerular filtration rate; M, month; NS, nonsignificant *P*; x-ULN, x-time the upper limit of normal for age; SDS, SD score.

Wilcoxon non-parametric test for quantitative variables or Fisher exact test for calculating the proportion of patients receiving hyperhydration/citrate/pyridoxine, etc..

### DAILY-C Subcohort (*n* = 10)

**Figure 5:**
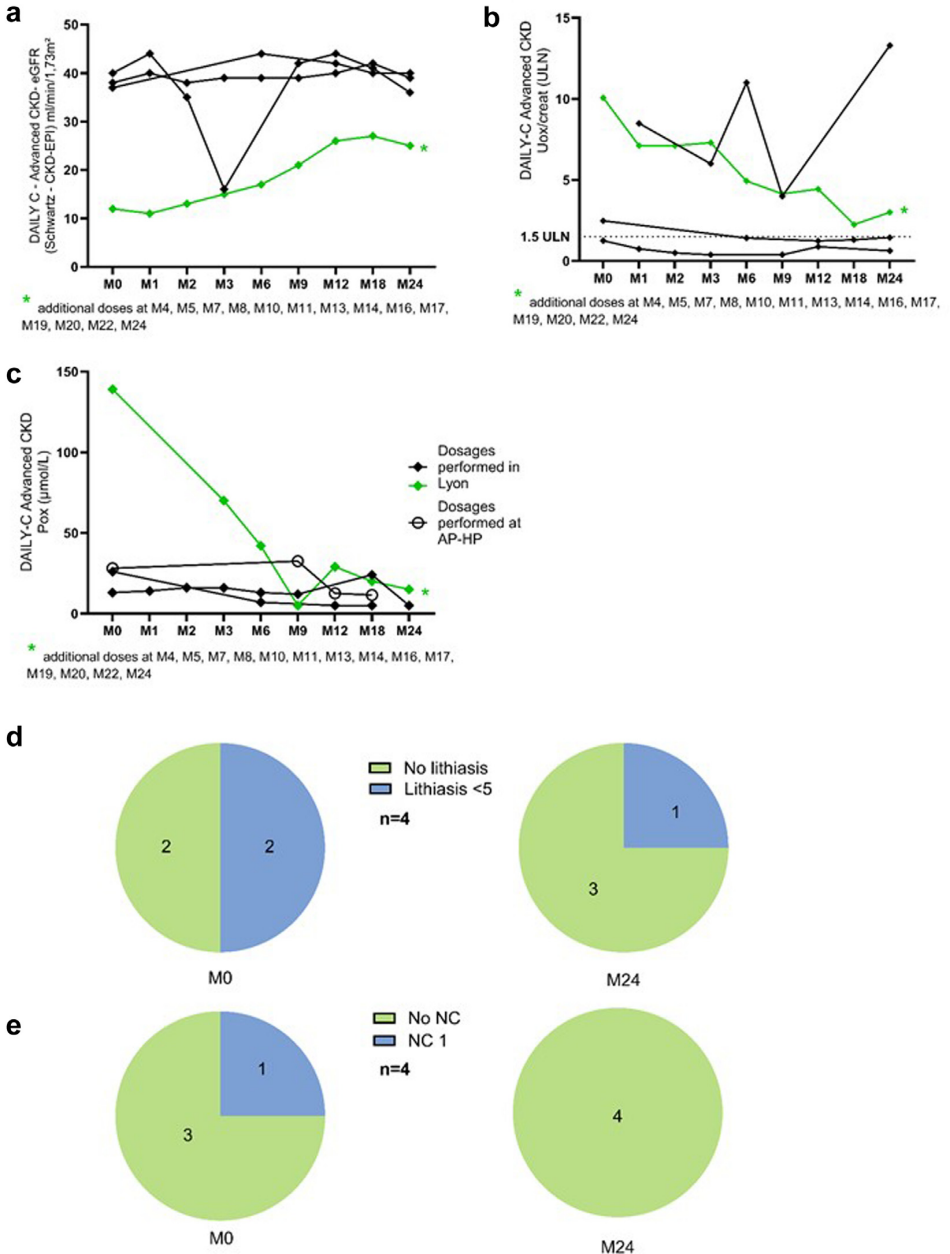
DAILY-C – advanced CKD (*n* = 4). (a) eGFR trends in nondialysis patients. (b) UOx/creat trends. (c) POx trends in nondialysis patients. UOx trends in nondialysis patients. (d) Change in lithiasis numbers in nondialysis patients on ultrasound imaging. (e) Change in nephrocalcinosis in nondialysis patients on ultrasound imaging. The evaluation of patient in green may seem atypical: this patient was 3 months of age at lumasiran initiation, and the trends of POx levels paralleled the trends of eGFR. There was no lithiasis but bilateral nephrocalcinosis stage 1 from M0 to M6 of therapy; nephrocalcinosis disappeared at M9. UOx/creatinine displayed as xx-times the ULN for age. CKD-EPI, Chronic Kidney Disease Epidemiology Collaboration; eGFR, estimated glomerular filtration rate; M, month; NC, nephrocalcinosis; POx, plasma oxalate; ULN, upper limit for normal; UOx/creat, urinary oxalate-to-creatinine ratio.

In this subgroup, 6 patients were on dialysis (4 on hemodialysis [HD], and 2 on peritoneal dialysis [PD]; 3 patients with infantile oxalosis). The 4 patients on conservative therapy with advanced CKD were diagnosed at a median age of 7.2 (0.3–32.0) years, with an initiation of lumasiran at 17.5 (0.3–51.0) years. The trends in the 4 patients on conservative therapy are displayed in Figure 5; no statistical analyses were performed in this subgroup because of the low number of patients. Of note, 3 patients had an eGFR at lumasiran initiation between 30 and 40 ml/min per 1.73 m^2^, with a stabilization over time (none of the patients progressed toward kidney failure); the patient with an eGFR of 12 ml/min per 1.73 m^2^ at initiation progressively recovered kidney function, with an eGFR of 25 ml/min per 1.73 m^2^ after 2 years: this patient was aged 3 months at lumasiran initiation, and the trends of POx levels paralleled the trends of eGFR, from 139 to 15 μmol/l.

**Figure 6:**
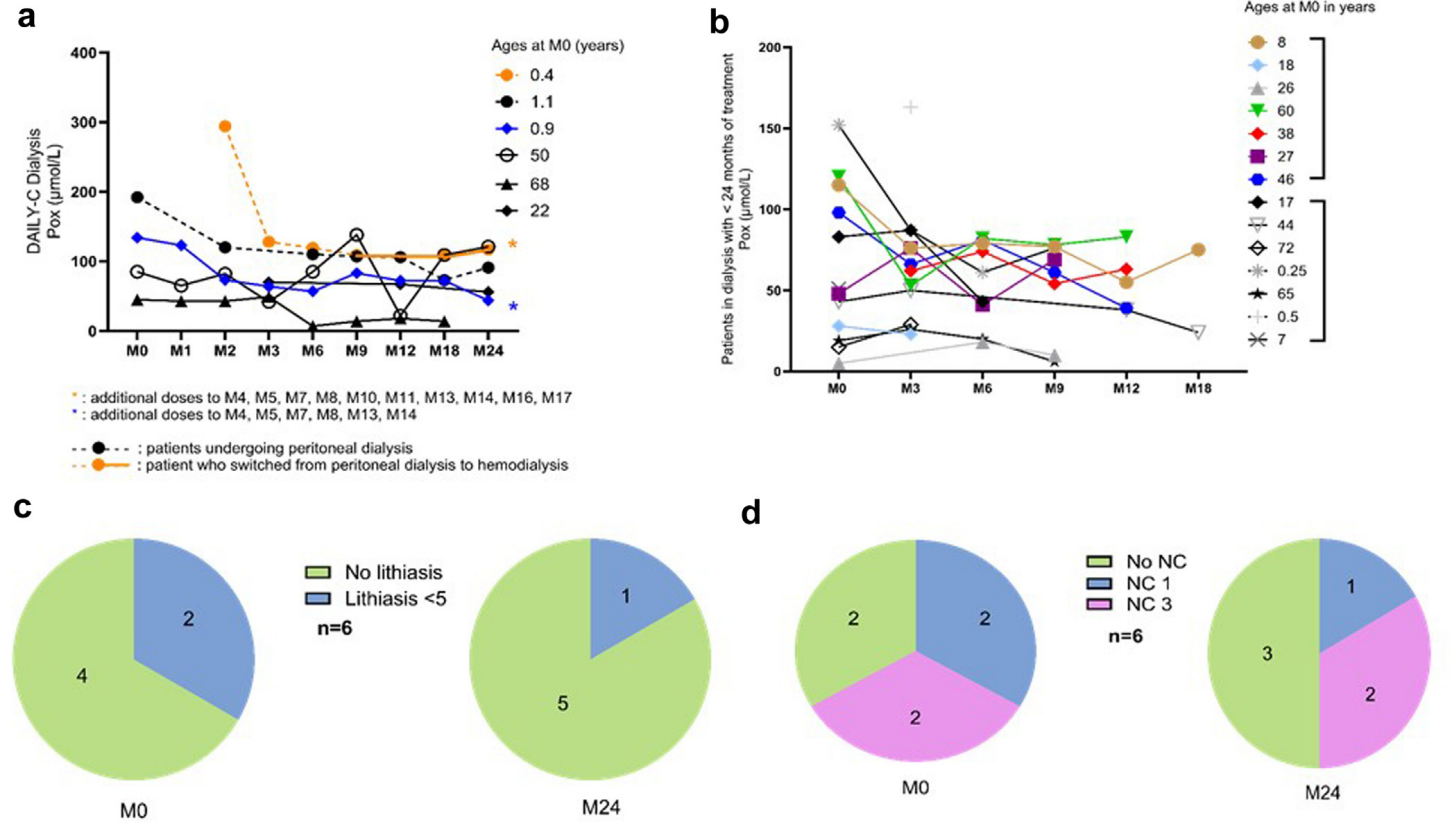
DAILY-C dialysis patients. (a) POx trends in dialysis. (b) POx trends in (1) patients who begun lumasiran in dialysis and who were transplanted before 2 years of treatment (the last POx on the graph corresponds to the last POx available in the medical charts before transplantation) (*n* = 7), and (2) patients who begun lumasiran in dialysis currently before 2 years of treatment (the last POx on the graph corresponds to the last follow-up) (*n* = 7). (c) Change in lithiasis numbers on ultrasound imaging. (d) Change in nephrocalcinosis on ultrasound imaging. M, month; Pox, plasma oxalate.

In dialysis, 2 patients (aged 5 and 13 months) were receiving peritoneal dialysis only, with a less satisfying control of POx levels, the plateau being around 110 μmol/l, whereas it was lower in the other 4 patients on HD. Even among patients on dialysis, there was a trend toward an improvement of nephrocalcinosis severity and number of stones. No metabolic acidosis was reported; there were no changes in circulating bicarbonate levels and no decrease in the prescription of citrate (that was prescribed over time in only 1 patient). Patients on HD were on intensive regimens at lumasiran initiation, and remained on intensive regimens. They had no significant changes in the number of dialysis hours per week, with a median number of 22 hours of HD per week at lumasiran initiation and 18 hours at 2 years. One patient switched from PD to HD after 9 months of lumasiran. Two patients died while on dialysis, 1 from Ear Nose Throat cancer and 1 from skin cancer. These results are displayed in Figure 6 and Table 4; because the number of patients on dialysis was low, we have added an additional panel considering the 7 patients who initiated lumasiran while on dialysis and who received a transplantation before 2 years of lumasiran in dialysis (Figures 1 and 6); of note 2 patients received a transplantation before reaching dialysis. These patients were older than the ones included in DAILY-C, and their POx levels were lower.

**Table 4:** Results in DAILY-C (only dialysis patients)

*N* = 6	Parameters	M0 (*n* = 6)	M1 (*n* = 6)	M2 (*n* = 6)	M3 (*n* = 6)	M6 (*n* = 6)	M9 (*n* = 6)	M12 (*n* = 6)	M18 (*n* = 6)	M24 (*n* = 6)	*P*-value (between M0 and M24)
Clinical characteristics at M0	Age (yrs)	11.5 (0.5–68.0)									
	Age at diagnosis (yrs)	9.8 (0.3–64.0)									
	Age at intensive dialysis initiation (yrs)	9.0 (0.3–64.0)									
	Body weight (kg)	32.0 (4.0–86.0)								34.7 (11.5–80.0)	ns
	Body weight (SDS)	−0.8 (−4.9 to 1.8)								−0.8 (−1.8 to 1.5)	ns
	Height (cm)	115.3 (60.0–172.0)								123.3 (85.0–172.0)	ns
	Height (SDS)	−0.4 (−2.2 to 0.4)								−1.1 (−2.7 to 0.7)	ns
	Residual diuresis (*n*)	1	1	1	1	1	1	1	1	1	
	Enlisted on renal transplant list (*n*)	2	2	2	2	2	2	2	3	3	
	Enlisted on double kidney-liver transplant list (*n*)	1	1	1	1	1	1	1	1	1	
	Number of fractures within the last 12 mo							2 patients with 3 fractures		1 patient with 1 fracture	
Management	K-Na citrate (*n*)	1	1	1	0	0	0	0	0	0	ns
	Pyridoxine (*n*)	5	5	5	4	4	4	4	4	3	ns
	G-tube (*n*)	3	3	3	3	3	3	3	3	3	ns
	HD/PD (*n*)	4–2	4–2	4–2	4–2	4–2	5–1	5–1	5–1	6–0	
	If HD, number of sessions per wk	6	6	6	6	6	6	6	6/wk in 4 patients, and 5/wk in 1	6/wk in 4 patients, and 5/wk in 1	
	If HD, dialysis h/wk	22.5 (18.0–24.0)	22.5 (18.0–24.0)	22.5 (18.0–24.0)	22.5 (18.0–24.0)	22.5 (18.0–24.0)	18.0 (18.0–24.0)	18.0 (18.0–24.0)	18.0 (18.0–24.0)	18.0 (18.0–24.0)	
	ESA (*n*)	4	4	4	4	4	4	4	4	4	
	ESA dose (UI/kg/wk)	207 (81–787)	197 (80–787)	219 (100–778)	196 (163–645)	186 (162–729)	381 (131–686)	458 (137–625)	443 (143–609)	524 (143–696)	
	Number of transfusions within the last 12 mo	4 patients received 3.5 (1.0–5.0) transfusions						1 patient received 5 transfusions the first yr		The same patient received 2 additional transfusions between 12 and 24 mo	
Biomarkers	Bicarbonate (mmol/l)	24 (19–27)	24 (20–32)	24 (15–26)	24 (23–25)	22 (22–29)	25 (20–35)	23 (21–28)	25 (21–27)	23 (18–30)	ns
	Hemoglobin (g/l)	100 (87–104)	95 (82–104)	104 (87–116)	94 (81–106)	99 (86–122)	103 (89–112)	105 (102–138)	106 (96–137)	111 (73–146)	ns
	Calcium (mmol/l)	2.39 (2.07–2.68)	2.45 (2.27–2.87)	2.33 (2.20–2.49)	2.39 (2.35–2.51)	2.49 (2.00–2.51)	2.51 (2.24–2.68)	2.34 (1.36–2.54)	2.32 (1.27–2.47)	2.26 (1.16–2.34)	ns
	Phosphate (mmol/l)	1.93 (0.81–2.68)	1.00 (0.96–2.89)	2.06 (1.14–4.28)	1.78 (1.41–2.14)	1.32 (0.77–2.50)	1.37 (1.30–1.79)	1.78 (0.73–1.90)	1.13 (0.79–1.50)	1.16 (0.68–1.90)	ns
	25OHD (nmol/l)	83 (38–165)	121–280	33–111	40–131	70–133	130 (88–329)	103 (42–113)	98	58 (42–116)	ns
	PTH (ng/l)	181 (23–350)	251 (71–335)	111–905	208 (161–529)	130–463	255 (15–506)	408 (178–597)	35–121	243 (48–486)	ns
	POx (μmol/l)	110 (45–192)	65 (43–123)	82 (43–294)	64 (42–128)	85 (7–119)	107 (14–138)	70 (18–106)	73 (14–109)	91 (44–121)	ns

25 OHD, 25-hydroxy vitamin D; eGFR, estimated glomerular filtration rate; ESA, erythropoietin-stimulating agent; HD, hemodialysis; M, month; NS, nonsignificant *P*;PD, peritoneal dialysis;PTH, parathyroid hormone; SDS, SD score; x-ULN, x-time the upper limit of normal for age.

Wilcoxon non-parametric test for quantitative variables or Fisher exact test for calculating the proportion of patients receiving hyperhydration/citrate/pyridoxine, etc.

All POx were assessed in the same reference laboratory in Lyon for these patients.

### Safety

Tolerance was good with no severe side effects. Injection site reactions, abdominal pain, and headaches were the main reported adverse events. In Supplementary Table S2, we summarize all reported side effects over time. Of note, 1 patient with infantile oxalosis on PD developed unexplained and clinically asymptomatic antineutrophil cytoplasmic autoantibody antibodies with hypereosinophilia, that were stable over time with no clinical symptoms; lymphocyte subpopulations were normal.

## Discussion

DAILY-LUMA is the first nationwide cohort reporting the use of lumasiran in a daily clinical setting, with almost-full exhaustivity at a national level (i.e., 92% of patients receiving lumasiran in France were included in the analysis). A previous retrospective study has reported outcomes in 33 patients (20 with preserved kidney function and 13 on dialysis) with PH1 receiving lumasiran for a median time of 18 months from 4 different European countries (Spain, Germany, Greece, and Hungary)^17^; however, national exhaustivity was not achieved, likely inducing selection bias from expert centers interested in PH1. Our national data confirm the efficacy and safety of lumasiran in patients with PH1, outside industry-sponsored trials in which the global management is tightly controlled in selected patients. The underlying genotypes in our patients confirm the representativity of genotypes previously described.^18^ Some patients in DAILY-A and DAILY-C started lumasiran with UOx or POx lower than in ILLUMINATE-A (mean 24-hour UOx ≥ 0.70 mmol/24 h per 1.73 m^2^) and ILLUMINATE-C (POx ≥20 μmol/l), thus illustrating the gap between clinicals trials and real life, with the potential to even improve long-term outcomes in treated patients with “milder” PH1 forms.

By analyzing patients similar to what was done in the ILLUMINATE-A and ILLUMINATE-B studies,^7^^,^^10^ we show similar patterns of response to lumasiran in terms of UOx trends in DAILY-A and DAILY-B. However, even though the primary endpoint in ILLUMINATE -A was derived from 24-hour urine collection,^7^ and that the 2023 European guidelines highlighted the importance of 24-hour urine collection,^3^ we show here that there is a gap between guidelines and real life. Indeed, most patients (pediatric and adults) are followed-up only with UOx/creat ratio in spot urine, despite the variability of such an assessment. In total, we only have 26 24-hour urinary samples, thus rendering impossible the use of this data in the manuscript. Other markers of efficacy, such as eGFR stabilization, decreased severity of nephrocalcinosis, and decreased number of stones, are similar to the results reported in the industry-sponsored trials. By describing individual trajectories of UOx/creat ratio and eGFR, we highlight relevant clinical points: the patients with the highest UOx/creat ratio at baseline displayed the most important relative decrease in UOx/creat ratio; this is very important because there is a clear association between baseline UOx and the risk of subsequent kidney failure.^15^ Likewise, the stabilization (and even improvement in the youngest patient) of eGFR in patients with the lowest eGFR at baseline (i.e., about 50–60 ml/min per 1.73 m^2^) is promising for (very) long-term outcomes and will deserve specific attention when long-term data at 5 years are available. The favorable trends in the eGFR in pediatric cases, more than in the older subgroups, is also likely because of renal maturation, as this is also the case for one patient in DAILY-C, receiving lumasiran from 3 months of age, with a dramatic increase of eGFR from 12 to 25 ml/min per 1.73 m^2^ during the 2-year follow-up. The same goes for the decreased severity of nephrocalcinosis in the youngest patients, as also previously reported,^19^ thus providing an additional rationale to treat these infants early. It is nevertheless interesting to note that the change in nephrocalcinosis in the pediatric DAILY-A subgroup is not so rapid and will need a longer follow-up. The absence of nephrocalcinosis at lumasiran initiation in adults from DAILY-A is intriguing but was double-checked with each treating physician when analyzing the data. Even though we were not able to directly assess the change in the quality of life in DAILY-A and DAILY-B, indirect markers such as the decrease in the proportion of patients with nocturnal hydration and G-tubes likely reflects improved quality of life for both patients and families. The same goes for the decreased number of stones.

In DAILY-B, it is interesting to note that 3 of 6 patients have received additional doses of lumasiran: as previously reported by us and others,^17^^,^^20^ these patients with body weight of about 10 kg seem to need lumasiran more often. The protocol of lumasiran administration depends on body weight and was arbitrarily decided by the pharmaceutical company based on theorical data on hepatic immaturity. Hepatic weight as a fraction of body weight is significantly higher in infants and decreases with growth, and this can lead to a faster hepatic metabolism in infants and quicker medication elimination than in older children.^21^ Here, this decision of increasing the number of injections was at the discretion of the treating physician when they considered that response to lumasiran was considered “inadequate,” whether based on UOx or POx depending on CKD stage, and allowed a satisfying change of UOx/creat ratio, and no side effect was observed.

This point should be kept in mind by pediatric nephrologists taking care of such patients, provided that their national regulations allow them to prescribe “off-label” doses of lumasiran.

The results obtained in the DAILY-C subgroup are intriguing and seem less favorable than the data published in ILLUMINATE-C,^9^ at least in patients on dialysis. Considering that it is well-known that there is a variability in POx results depending on the laboratories in which it is measured, we first ruled out this explanation, because all POx of the patients on dialysis were measured in the reference center of Lyon. Second, and this is the main difference with ILLUMINATE-C (in which investigators could not change the dialysis regimen for 12 months); we evaluated whether the introduction of an RNAi therapy was associated with a decrease in the weekly frequency of dialysis regimens. Indeed, even though conceptually, it is important to maintain intensive dialysis strategies under lumasiran to clear as much as possible oxalate storage from the bone (RNAi therapies will have no effect on this component of systemic oxalosis because they only block the hepatic oxalate synthesis) before transplantation,^3^^,^^12^^,^^14^ it may be possible that in real life, the shared decision with the patient leads to less intensive regimens to improve their quality of life. However, we do not have evidence that this was the case here. Interestingly, 3 of 6 patients had infantile oxalosis, among them 2 receiving only PD, with a less satisfying control of POx levels; this could be expected from older studies on oxalate clearance in PD showing decreased oxalate dialysance in PD.^22^ Infantile oxalosis is well-known to be the most severe form of PH1.^4^ In the ILLUMINATE-C trial, the youngest treated patient was 1-year old. The proportion of infantile oxalosis was likely lower in the industry-sponsored trial.^9^ Moreover, here, only 17% of patients had a residual diuresis. This proportion is not known in ILLUMINATE-C; however, the presence of residual diuresis in dialysis is associated with decreased POx levels (personal data). With all these data in mind, the most likely cause of these results in DAILY-C is nevertheless the proportion of patients with infantile oxalosis. Indeed, older patients undergoing dialysis who were transplanted before 2 years of follow-up in dialysis had lower POx levels, and a change in POx levels mimicking the one described in ILLUMINATE-C.^9^

In any case, dialysis in PH1 should last for the shortest possible duration, to avoid the worsening of systemic oxalosis and to propose transplantation strategies.^3^ In that setting, at the end of the follow-up period in dialysis for the DAILY-C subgroup, only 2 patients had POx levels < 72 μmol/l, corresponding to the highest POx levels reported safe for isolated kidney transplantation under lumasiran.^14^ The question of better evaluation of systemic oxalosis (as opposed to POx levels) remains open: as already suggested in a previous publication from our group,^12^ the decreased need of blood transfusions in patients on dialysis receiving lumasiran may indicate an improvement of systemic oxalosis. We could have expected a decrease in erythropoietin-stimulating agent dose. However, even though it does not reach statistical significance, hemoglobin levels are 1 g/l higher after 2 years of follow-up, and patients received less blood transfusions. The occurrence of fracture in dialysis is not surprising even under RNAi. Indeed, knowledge from combined liver or kidney transplantation indicates that it takes time after transplantation to get rid of all oxalate trapped mainly in bone,^23^ thereby increasing bone fragility during oxalate release from bone.^24^ Even in patients receiving RNAi, systemic oxalosis may occur, especially in dialysis. RNAi will have no effect on oxalate bone release, and, as showed by Garrelfs *et al.*, there is an intraindividual variation of glycolate oxidase inhibition by lumasiran in patients with PH1 from 55% to 91%.^25^ To better address systemic oxalosis, we also reviewed cardiac and ocular data, but the number of patients undergoing such evaluation was low (Supplementary Table S3).

Trying to address the concerns raised by Martin-Higueras *et al.* reporting a lumasiran-induced acid state in patients on dialysis that was buffered by increased dialysis regimen and citrate doses in their cohort,^17^ we did not find any direct or indirect evidence for metabolic acidosis in these patients. However, physicians should be aware of this potential side effect, because of its potential severity. Lastly, some patients on dialysis had dose adjustments, again mainly in the youngest ones, and following the ongoing amendment in the ILLUMINATE-C trial allowing to increase the lumasiran dose and/or frequency of administration in case of inadequate control of POx levels in dialysis.

The main strengths of the study are the almost full exhaustivity of included patients at a national level, the quality of the data because a single clinical research associate was in charge of all data collection, the granularity of data, and the number of patients with at least 2 years of follow-up in the field of an ultrarare disease. The limitations are mainly rooted in the nature of registry data as being retrospective in this first analysis. The methodology chosen here was to “copy-paste” the industry-sponsored trials, by using the similar inclusion criteria and the exact same follow-up (2 years) to compare first trials and real life. This is a methodological choice, and we should have much more data in the future with more patients, because the study is still ongoing to get 5 year-follow up in more than 90 patients.

In conclusion, the DAILY-LUMA study is currently the largest nationwide cohort of patients treated with lumasiran in real life. We confirm the safety and efficacy of lumasiran in PH1, notably in terms of decrease in oxalate levels, stabilization of renal function, and improvement or disappearance of kidney stones and nephrocalcinosis. We also illustrate the gap between guidelines and clinical routine for the follow-up of patients, and provide indirect evidence that the quality of life of patients and their families is likely improved. Long term-follow-up data at 5 years will be necessary to confirm these preliminary albeit promising data.

## Disclosure

JB received speaker and consulting fees from Alnylam, Dicerna/Novo-Nordisk, and Biocodex; and travel fees from Alnylam. A-LS-L received speaker and consulting fees from Alnylam and Dicerna/Novo-Nordisk. CA-B received speaker and consulting fees from Alnylam. OB received support for attending meetings and/or travel and consulting fees from Biocodex and Alnylam; and payment or honoraria for lectures, presentations, speakers’ bureaus, manuscript writing, or educational events from Alnylam. TK received support for attending meetings and/or travel from Hemotech, and participation on a Data Safety Monitoring Board or Advisory Board from Hemotech and Sanofi. SB received speaker and consulting fees from Astra-Zeneca, Amgen, Borhinger-Ingelheim, and CSL Vifor. TS received funding of travel, hotel, and registration to the annual congress of the French Society of Nephrology, Dialysis and Transplantation in 2022, from the Alnylam Laboratory. MC received grants or contracts from Advicienne; and consulting fees, payment or honoraria for lectures, presentations, speakers’ bureaus, manuscript writing or educational events and support for attending meetings and/or travel from Alnylam/Viatris. AS received support for attending meetings and/or travel from Theradial. All the other authors declared no competing interests.

## Data Availability Statement

The datasets generated during and/or analyzed during the current study are not publicly available because of confidential data but are available from the corresponding author on reasonable request.

## Supplementary Material

Supplementary File (PDF) **Table S1.** Underlying genotypes. **Table S2.** Side effects during follow-up. **Table S3.** Cardiac and ocular follow-up. STROBE Checklist.

## Footnotes

Supplementary File (PDF)
**Table S1.** Underlying genotypes. **Table S2.** Side effects during follow-up. **Table S3.** Cardiac and ocular follow-up. STROBE Checklist.
