# Supplementary material for: Real-Life Data of 2-Year Lumasiran Use in the DAILY-LUMA Cohort
Source: Kidney Int Rep. 2024 Dec 30;10(4):1020–36. doi: 10.1016/j.ekir.2024.12.033 (PMC12034872; doi:10.1016/j.ekir.2024.12.033)
Supplement: Supplementary File (PDF) — Table S1. Underlying genotypes. Table S2. Side effects during follow-up. Table S3. Cardiac and ocular follow-up. STROBE Checklist. [file mmc1.pdf]

Supplementary Material

- STROBE Checklist
- Supplemental Table 1: underlying genotypes
- Supplemental Table 2: side effects during follow- up
- Supplemental Table 3: cardiac and ocular follow-up

Modified STROBE Statement—checklist of items that should be included in reports of observational studies (Cohort/Cross-sectional and case-control studies)

	Item No	Recommendation	Page
**Title and abstract**	1	(*a*) Indicate the study's design with a commonly used term in the title or the abstract	Page 3 lines 7-8
		(*b*) Provide in the abstract an informative and balanced summary of what was done and what was found	Page 3 lines 14- 24
**Introduction**			
Background/rationale	2	Explain the scientific background and rationale for the investigation being reported	Page 4 lines 2-20
Objectives	3	State specific objectives, including any prespecified hypotheses	Page 5 lines 8-10
**Methods**			
Study design	4	Present key elements of study design early in the paper	Page 5 lines 13-18
Setting	5	Describe the setting, locations, and relevant dates, including periods of recruitment, exposure, follow-up, and data collection	Page 6 lines 1- 21
Participants	6	(*a*) *Cohort study*—Give the eligibility criteria, and the sources and methods of selection of participants. Describe methods of follow-up<br><br>*Case-control study*—Give the eligibility criteria, and the sources and methods of case ascertainment and control selection. Give the rationale for the choice of cases and controls<br><br>*Cross-sectional study*—Give the eligibility criteria, and the sources and methods of selection of participants	Page 7 lines 3-17
Variables	7	Clearly define all outcomes, exposures, predictors, potential confounders, and effect modifiers. Give diagnostic criteria, (if applicable)	Page 6 lines 2- 21
Data sources/ measurement	8*	For each variable of interest, give sources of data and details of methods of assessment (measurement).	Page 6 lines 18 - page 7 line 6

Bias	9	Describe any efforts to address potential sources of bias	Page 7 lines 3-6
Study size	10	Explain how the study size was arrived at (if applicable)	Page 7 lines 7-17
Quantitative variables	11	Explain how quantitative variables were handled in the analyses. If applicable, describe which groupings were chosen and why	Page 7 lines 18-21
Statistical methods	12	(*a*) Describe all statistical methods, including those used to control for confounding	Page 7 lines 18-21
		(*b*) Describe any methods used to examine subgroups and interactions	Page 7 lines 7-17
		(*c*) Explain how missing data were addressed	Page 8 lines 5- 10
		(*d*) *Cohort study*—If applicable, explain how loss to follow-up was addressed<br>*Case-control study*—If applicable, explain how matching of cases and controls was addressed<br>*Cross-sectional study*—If applicable, describe analytical methods taking account of sampling strategy	Page 8 lines 5-6
		(*e*) Describe any sensitivity analyses	
**Results**			
Participants	13*	(a) Report numbers of individuals at each stage of study—eg numbers potentially eligible, examined for eligibility, confirmed eligible, included in the study, completing follow-up, and analyzed	Page 8 lines 1-5
		(c) **Use of a flow diagram**	Figure 1
Descriptive data	14*	(a) Give characteristics of study participants (eg demographic, clinical, social) and information on exposures and potential confounders	Page 8 lines 12-13; Table 1<br>Page 9 lines 3-4 ; Table 2<br>Page 9 lines 18-19; table 3<br>Page 10 lines 9-12
		(b) Indicate number of participants with missing data for each variable of interest	Page 8 lines 5-10
		(c) *Cohort study*—Summarise follow-up time (eg, average and total amount)	Page 8 lines 2-4

Outcome data	15*	*Cohort study*—Report numbers of outcome events or summary measures over time	Page 8 lines 15-25 page 9 lines 1-2<br>Page 9 lines 3-16<br>Page 9 lines18-25 page 10 lines 1-7<br>Page 10 lines 9-19<br>Page 10 lines 20-25 page 11 lines1-9
		Case-control study—Report numbers in each exposure category, or summary measures of exposure	N.A.
		Cross-sectional study—Report numbers of outcome events or summary measures	N.A.
Main results	16	(*a*) Give unadjusted estimates and, if applicable, confounder-adjusted estimates and their precision (eg, 95% confidence interval). Make clear which confounders were adjusted for and why they were included	N.A
Other analyses	17	Report other analyses done—eg analyses of subgroups and interactions, and sensitivity analyses	N.A
**Discussion**			
Key results	18	Summarise key results with reference to study objectives	Page 11 lines 18-19<br>Page 12 lines 1-2
Limitations	19	Discuss limitations of the study, taking into account sources of potential bias or imprecision. Discuss both direction and magnitude of any potential bias	Page 12 line 6-7<br>Page 12 line 13-16<br>Page 13 lines 13-14<br>Page 14 line 3<br>Page 14 line 11-13<br>Page 16 lines 3-11
Interpretation	20	Give a cautious overall interpretation of results considering objectives, limitations, multiplicity of analyses, results from similar studies, and other relevant evidence	Page 16 line 12-18

Generalisability	21	Discuss the generalisability (external validity) of the study results	Page 16 line 12-18.

*Give information separately for cases and controls in case-control studies and, if applicable, for exposed and unexposed groups in cohort and cross-sectional studies.

Note: An Explanation and Elaboration article discusses each checklist item and gives methodological background and published examples of transparent reporting. The STROBE checklist is best used in conjunction with this article (freely available on the Web sites of PLoS Medicine at http://www.plosmedicine.org/, Annals of Internal Medicine at http://www.annals.org/, and Epidemiology at http://www.epidem.com/). Information on the STROBE Initiative is available at www.strobe-statement.org.

Supplemental Table 1: underlying genotypes

	**Genotypes *AGXT* (NM_000030)**	**Age at lumasiran initiation (years)**	**Age at diagnosis (years)**
**DAILY-A children (N=13)**			
04-001	c.508G>A/c.697C>T<br>p.Gly170Arg/p.Arg233Cys	15	1
04-002	c.508G>A/unidentified<br>p.Gly170Arg/?	13	0.9
04-006	c.33dup/c.1079G>A<br>p.Lys12Glnfs*156./p.Arg360Gln	17	4
05-001	c.976del/c.976del<br>p.Val326TyrfsX15/ p.Val326TyrfsX15	14	14
09-001	c.731C>T/c.731C>T<br>p.Ile244Thr/p.Ile244Thr	11	10
09-02	c.731C>T/c.731C>T<br>p.Ile244Thr/p.Ile244Thr	14	14
10-001	c.847- ?_1179+?del/ c.847-<br>?_1179+?del<br>p.?/p.?	7	2
13-001	c.976del/c.976del<br>p.Val326TyrfsX15/ p.Val326TyrfsX15	6	0
14-001	c.167T>A/c/167T>A<br>p.Ile56Asn/ p.Ile56Asn	18	4
15-001	c.547G>A/c.846+1G>T<br>p/Asp183Asn/p.?	15	7
17-001	c.525-1G>A/c.900dup<br>p.?/p/Arg301AlafsX31	6	6
18-001	c.454T>A/c.731T>C<br>p.Phe152Ile/p.Ile244Thr	12	5
28-001	c.358+2T>C/c.454T>A<br>p.?/p.Phe152Ile	11	3
**DAILY-A adults (N=9)**			
03-001	c.568G>A/c.568G>A<br>p.Gly190Arg/p.Gly190Arg	41	38
03-002	c.568G>A /c.731C>T<br>p.Gly190Arg/p.Ile244Thr	28	9
03-003	c.731C>T/c.731T>C<br>p.Ile244Thr/p.Ile244Thr	55	14
03-004	c.731C>T/c.731T>C<br>p.Ile244Thr/p.Ile244Thr	51	51
06-002	c.731C>T/c.731T>C<br>p.Ile244Thr/p.Ile244Thr	19	5
07-001	c.731C>T/c.731T>C<br>p.Ile244Thr/p.Ile244Thr	39	39
12-001	c.508G>A/c.847-3C>G<br>p.Gly170Arg/p.?	30	22
16-001	c.508G>A/c.969-1G>C<br>p.Gly170Arg/p.?	23	23
23-001	c.847-1G>C/c..1079G>A<br>p.?/p.Arg360Gln	28	25
**DAILY-B (N=6)**			
01-004	c.731C>T/c.731T>C<br>p.Ile244Thr/p.Ile244Thr	2	5.3
01-006	c.731C>T/c.731T>C<br>p.Ile244Thr/p.Ile244Thr	1	1,9
01-007	c.33dup/c.321_344del<br>p.Lys12Glnfs*156/p.Trp108_Ile115del	0.5	0
01-008	c.731C>T/c.731T>C<br>p.Ile244Thr/p.Ile244Thr	0.3	0,3
17-002	c.242C>T/c.508G>A<br>p.Ser81Leu/p.Gly170Arg	1	1
25-001	c.454T>A/c.697C>T<br>p.Phe152Ile/p.Arg233Cys	0.3	3.9

**DAILY-C Advanced CKD (N=4)**			
05-005	c.508G>A/c.508G>A p.Gly170Arg/p.Gly170Arg	2	0.3
07-002	c.731C>T/c.731T>C p.Ile244Thr/p.Ile244Thr	33	14
09-003	c.731C>T/c.731T>C p.Ile244Thr/p.Ile244Thr	51	32
26-001	c.647-1G>A/c.846G>C p. ?/p.Gln282His	0.25	0.25
**DAILY-C dialysis (N=6)**			
01-05	c.33dup/c.321_344del p.Lys12Glnfs*156/p.Trp108_Ile115del	1	0.5
03-005	c.33dup/c.33dup p.Lys12Glnfs*156/ p.Lys12Glnfs*156	22	19
08-002	c.731C>T/c.731T>C p.Ile244Thr/p.Ile244Thr	68	64
08-004	c.976del/c.976del p.Val326TyrfsX15/ p.Val326TyrfsX15	50	50
10-002	c.662_664del/c662_664del p.Ser211del/p.Ser211del	0.5	0.3
18-002	c.32_33del/c.731T>C p.Pro11GlnfsX156/p.Ile244Thr	1	0.3

Supplemental Table 2: side effects during follow- up

		M0	M1	M2	M3	M6	M9	M12	M18	M24
**Total cohort N=38**	**Injection site reaction (n)**	4	2	2	1	2	0	0	1	1
	**Nausea (n)**	1	0	1	0	0	0	0	0	1
	**Headaches (n)**	1	1	1	0	0	0	0	0	1
	**Abdominal pain (n)**	1	0	0	1	2	1	1	1	1
	**Vomiting (n)**	0	0	0	0	0	0	0	0	0
**DAILY-A children N=13**										
	**Injection site reaction (n)**	1	1	0	0	1	0	0	0	1
	**Nausea (n)**	0	0	0	0	0	0	0	0	0
	**Headaches (n)**	0	1	1	0	0	0	0	0	0
	**Abdominal pain (n)**	0	0	0	0	0	0	0	1	0
	**Vomiting (n)**	0	0	0	0	0	0	0	0	0
**DAILY-A adults N=9**										
	**Injection site reaction (n)**	1	0	0	0	1	0	0	0	0
	**Nausea (n)**	1	0	2	0	0	0	0	0	1
	**Headaches (n)**	1	0	0	0	0	0	0	0	0
	**Abdominal pain (n)**	0	0	0	0	0	0	0	0	0
	**Vomiting (n)**	0	0	0	0	0	0	0	0	0
**DAILY-B N=6**										
	**Injection site reaction (n)**	1	0	0	0	0	0	0	0	0
	**Nausea (n)**	0	0	0	0	0	0	0	0	0
	**Headaches (n)**	0	0	0	0	0	0	0	0	1
	**Abdominal pain (n)**	0	0	0	1	2	1	1	0	1
	**Vomiting (n)**	0	0	0	0	0	0	0	0	0
**DAILY-C Advanced CKD N=4**										
	**Injection site reaction (n)**	1	1	2	1	0	0	0	0	0
	**Nausea (n)**	0	0	0	0	0	0	0	0	0
	**Headaches (n)**	0	0	0	0	0	0	0	0	0
	**Abdominal pain (n)**	1	0	0	0	0	0	0	0	0
	**Vomiting (n)**	0	0	0	0	0	0	0	0	0
**DAILY-C dialysis N=6**										
	no side effects									

Supplemental Table 3: cardiac and ocular follow-up

Results presented as number of abnormal results / number of performed examinations	**Number of patients with abnormal cardiac US at baseline**	**Number of patients with abnormal cardiac US between M18 and M24**	**Number of patients with abnormal fundus examination at baseline**	**Number of patients with abnormal fundus examination between M18 and M24**
**DAILY-A kids (N=13)**	0/2	0/0	0/2	0/0
**DAILY-A adults (N=9)**	0/3	0/1	0/2	0/0
**DAILY-B (N=6)**	0/0	0/0	0/0	0/1
**DAILY-C (N=10)**	2/6	2/4	2/6	3/4
